# P_ion_ effects in flattening filter‐free radiation beams

**DOI:** 10.1120/jacmp.v16i6.5869

**Published:** 2015-11-08

**Authors:** Robert A. Corns, Vicky W. Huang, Steven D. Thomas

**Affiliations:** ^1^ Department of Medical Physics BC Cancer Agency — Fraser Valley Centre Surrey BC; ^2^ Department of Medical Physics BC Cancer Agency — Vancouver Centre Vancouver BC Canada

**Keywords:** flattening filter‐free, FFF, ion recombination, P_ion_

## Abstract

Flattening filter‐free radiation beams have higher dose rates that significantly increase the ion recombination rate in an ion chamber's volume and lower the signal read by the chamber‐electrometer pair. The ion collection efficiency correction ($P_{\text{ion}}$) accounts for the loss of signal and subsequently changes dosimetric quantities when applied. We seek to characterize the changes to the percent depth dose, tissue maximum ratio, relative dose factor, absolute dose calibration, off‐axis ratio, and the field width. We measured $P_{\text{ion}}$ with the two‐voltage technique and represented $P_{\text{ion}}$ as a linear function of the signal strength. This linear fit allows us to correct measurement sets when we have only gathered the high voltage signal and to correct derived quantities. The changes to dosimetric quantities can be up to 1.5%. Charge recombination significantly affects percent depth dose, tissue maximum ratio, and off‐axis ratio, but has minimal impact on the relative dose factor, absolute dose calibration, and field width.

PACS number: 87.55.N‐

## I. INTRODUCTION

Flattening filter‐free (FFF) beams offer superior dose rates for radiotherapy treatments than flattened beams. Field sizes less than $6\times 6\;{\text{cm}}^{2}$ are as flat as the flattened beams, making the FFF beam ideal for 3D conformal planning techniques that utilize small fields, such as stereotactic ablative radiotherapy (SABR). Larger fields are not flat, but the nonuniformity can be overcome with IMRT or VMAT techniques. Special attention needs to be paid to the FFF beams as recommended by AAPM Therapy Emerging Technology Assessment Work Group.^(1)^ The report discusses considerations unique to FFF beams in facility planning, commissioning, QA programs, treatment planning systems, treatment delivery, patient specific QA, practical clinical limitations, and safety. In this study, we are focusing on the ion recombination effects. Increasing the dose per pulse increases the ion recombination rate in an ion chamber's volume. The increased ion recombination rate decreases an ion chamber's collection efficiency. We correct for this reduction in our chamber's reading by using the collection efficiency correction, $P_{\text{ion}}$.

Several groups have studied FFF beams^2^, ^3^, ^4^ and concluded $P_{\text{ion}}$ significantly changes the percent depth dose (%dd) and off‐axis ratio (OAR) by 1% to 2%. When we commissioned our 6 FFF and 10 FFF beams, we realized $P_{\text{ion}}$ corrections also affect the tissue maximum ratio (TMR), the relative dose factor (RDF), the absolute dose rate, and the field widths.

One problem with $P_{\text{ion}}$ corrections is that the technique for measuring and applying these corrections is not compatible with how some dosimetric functions are acquired. For example, the %dd measurement uses dose rate as a surrogate for the dose and moves the chamber continuously. This is incompatible with $P_{\text{ion}}$ measurements, which require measuring the dose at a fixed location for two different voltages. Our solution was to measure $P_{\text{ion}}$ separately as a function of the ion chamber's signal strength and use this empirical function to correct the %dd curves.

Several groups have considered $P_{\text{ion}}$ as a function of the dose per pulse,^5^, ^6^ while others^7^, ^8^ have measured $P_{\text{ion}}$ in flattened radiation beams and characterized it in terms of depth and field size. We used the uncorrected signal with a normalization convention closely related to the dose per pulse. We normalized the electrometer's uncorrected signal using the reading for a $10\times 10\;{\text{cm}}^{2}$ field at the isocenter and at depth of dose maximum as a reference.

The application of $P_{\text{ion}}$ corrections to measured TMR values is simpler than for the %dd curves because TMR measurements are dose‐based and can be done together with $P_{\text{ion}}$ measurements. However, TMR measurements are impractical and people typically derive TMR curves from %dd curves. A number of software packages convert %dd to TMR, but the software will not correct the readings when computing the %dd and TMR. Consequently, some care is required when applying $P_{\text{ion}}$ corrections to the TMR curves.

The RDF measurement relates dose rate changes to field size. This, too, is a dose‐based measurement and $P_{\text{ion}}$ can be measured with each point in the RDF curve.

The TG‐51 protocol^(9)^ describes how to measure $P_{\text{ion}}$ and correct the reading. The protocol does not consider correcting the beam quality for $P_{\text{ion}}$ effects, although it does acknowledge such an effect exists but is not significant for electron fields. The absolute dose rate is proportional to the quality conversion factor $k_{Q}$ and because $k_{Q}$ depends on the %dd, anything that influences the %dd will change the absolute dose rate. We expected the impact $P_{\text{ion}}$ has to be small on absolute dose calibration because $k_{Q}$ does not vary rapidly with %dd. Nevertheless, we wanted to evaluate the size of this effect. According to TG‐51 addendum,^(10)^ the $k_{Q}$ formulism is for beams with flattening filters only and ion chambers with high‐Z electrodes should not be used for FFF beams. However, AAPM Therapy Emerging Technology Assessment Work Group^(1)^ states $k_{Q}$ is acceptable for beams with or without a flattening filter, with a maximum error of 0.4%.

Finally, if $P_{\text{ion}}$ changes the OAR, it could conceivably change the field width. This effect is of concern because SABR applications have tight geometric constraints and changes to the field width are important to understand and quantify.

As a secondary investigation, we wanted to assess the difference $P_{\text{ion}}$ makes between flat beams and FFF beams using our methodology. Charge recombination has been studied extensively in the literature,^3^, ^5^, ^11^, ^12^, ^13^, ^14^ with authors focusing on either flat or nonflat beams. Kry et al.^(5)^ has considered both beam types and found the change ($P_{\text{ion}}-1$) to be two to four times higher for FFF beams than for flat beams.

## II. MATERIALS AND METHODS

We commissioned our 6 FFF and 10 FFF beams on a TrueBeam linear accelerator (Varian Medical Systems, Palo Alto, CA) using a Wellhöfer Scanditronix water tank (Scanditronix Wellhöfer North America, Bartlett, TN), a WP‐3840 Manual Water Phantom (CNMC Company, Nashville TN), Wellhöfer IC10 (Scanditronix Wellhöfer North America, Bartlett, TN), and Exradin A19 ion (Standard Imaging, Middleton, WI) chambers together with a PTW UNIDOSE E electrometer (PTW‐Freiburg, Freiburg, Germany).

$P_{\text{ion}}$ depends on the dose per pulse. We cannot directly control the dose per pulse coming out of the linac, but we can change the dose rate by either changing the monitor unit rate or by changing the location of our chamber relative to the source. The monitor unit rate does not actually change the dose per pulse, but rather changes the number of beam pulses per unit time. We confirmed $P_{\text{ion}}$ is independent of the monitor unit rate by measuring it with a $10\times 10\;{\text{cm}}^{2}$ field and the ion chamber placed in phantom at depth‐of‐dose maximum in a source‐to‐axis distance (SAD) setup. For the remainder of the measurements, we used both SAD and source‐to‐surface distance (SSD) setups and varied the energy, distance, depth, off‐axis position, and field size to change the dose per pulse.

As a convention to know when the $P_{\text{ion}}$ corrections have been used, we will use a superscripted asterisk to denote values or functions that have not had $P_{\text{ion}}$ corrections. We measured $P_{\text{ion}}$ using Boag's two‐voltage technique.^3^, ^12^ Kry et al.^(5)^ confirmed for FFF beams the validity of the two‐voltage method for determining $P_{\text{ion}}$ against the more accurate method of plotting Jaffé plots (1/V versus 1/Q curves). The two‐voltage method uses a ratio of raw electrometer readings $M_{\text{high}}^{*}$ and $M_{\text{low}}^{*}$ taken for the high and low voltage settings $V_{\text{high}}$ and $V_{\text{low}}$, respectively, and computes $P_{\text{ion}}$ as

(1) $$P_{ion}=\frac{1-(V_{high}/V_{low})}{\left(M^{*}{\;}_{high}/M^{*}{\;}_{low})-(V_{high}/V_{low}\right)}$$

By convention, our high and low voltage settings are 300 V and 150 V. The ratio of the electrometer readings is invariant against scaling of the readings and we decided to renormalize the signals to a standardized scale. Renormalizing allows easy comparison of $P_{\text{ion}}$ effects for different chambers that have different chamber/electrometer sensitivities for the same delivered MU.

Our reference scale assigns 100 units to a $10\times 10\;{\text{cm}}^{2}$ field with the chamber set up under SAD conditions at depth of dose maximum and the voltage set to 300 V. All other readings for this energy are scaled relative to this value, including those for the low voltage settings. Experimentally, we can do this by measuring a signal $M_{\text{ref}}^{*}$ for the reference $10\times 10\;{\text{cm}}^{2}$ field and a signal $M^{*}$ for the field of interest in some other setup. The renormalized signal $R^{*}$ is proportional to the ratio of the electrometer readings:

(2) $$S^{*}=100\frac{M^{*}}{M_{ref}^{*}}$$

The signal corrected for $P_{\text{ion}}$ is shown in Eq. (3). Here, we have left out $P_{\text{pol}},P_{\text{hum}},P_{\text{elec}}$ and $P_{\text{TP}}$ from the corrected meter readings because these factors cancel out when we take a ratio of our signal to the reference signal.

(3) $$S=P_{ion}S^{*}$$

This method is advantageous because the reference signal for each measurement run does not need to be measured. The $P_{\text{ion}}$ correction can be applied based on the magnitude of the renormalized signal, $M^{*}$.

We evaluated the effect of $P_{\text{ion}}$ on dosimetric functions by computing absolute and relative differences of the function with and without the $P_{\text{ion}}$ correction. For definiteness, consider the %dd. We compared the corrected %dd to the uncorrected %dd^*^ and took the absolute difference and the relative differences:

(4) $$\Delta_{\%dd\;abs}=\%dd-\%dd^{*}$$

(5) $$\Delta_{\%dd\;rel}=\frac{\%dd-\%dd^{\%}}{\%dd}$$

The change Δ depends on depth, field size, and energy. In addition, the crossplane position is important for $\Delta_{\text{OAR}}$.

The absolute output has an additional dependence on $P_{\text{ion}}$ because the quality conversion factor depends on the %dd. The absolute dose is given in the TG‐51 protocol as

(6) $$D_{w}^{Q}=Mk_{Q}N_{D,w}^{60_{Co}}$$

The impact of $P_{\text{ion}}$ on $D_{w}^{Q}$ is evaluated by the relative difference of $k_{Q}$ and $k_{Q}^{*}$.

The field width (w) is determined from the locations of the 50% isodoses in the beam profile where the central axis has been normalized to 100%. We computed the change in width, $\Delta_{w}$:

(7) $$\Delta_{w}=w-w^{*}$$

for $3\times 3\;{\text{cm}}^{2}$ to $6\times 6\;{\text{cm}}^{2}$ fields because these are most critical for 3D conformal SABR applications, and for a $20\times 20\;{\text{cm}}^{2}$ field to confirm the large field behavior.

We performed statistical analysis using JMP version 10.0.2 (2012, SAS Institute, Cary, NC), R version 3.0.1 (The R Foundation for Statistical Computing, 2013, https://www.r‐project.org/), OpenBUGs version 3.2.3 rev 1012 (2014, Members of the OpenBugs Project Management Group, http://www.openbugs.net/w/FrontPage), and JAGS version 3.4.0 (2013, GNU General Public License, http://mcmc‐jags.sourceforge.net/).

## III. RESULTS

**Figure 1:**
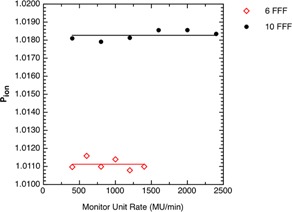
The ion recombination correction $P_{\text{ion}}$ plotted as a function of the monitor unit rate. The solid lines represent the mean $P_{\text{ion}}$ value for each energy.

Figure 1 presents the results of $P_{\text{ion}}$ measurements against the monitor unit rate. We fitted each dataset with a simple linear model using Bayesian analysis. Specifically, we wanted to know if the slope was believably different from 0. The 95% highest density intervals (HDI) for the slopes for the 6 FFF and 10 FFF beams are, respectively: (−0.000078, 0.000073) and (−0.000039, 0.000037). In each case, 0 is a credible slope because it is in the 95% HDI and we conclude $P_{\text{ion}}$ is independent of the monitor unit rate. As a consequence, we set the monitor unit rate to the maximum possible value for all other measurements.

**Figure 2:**
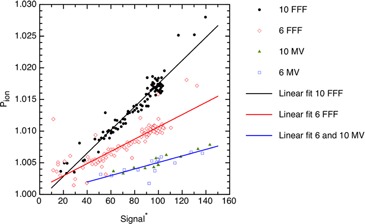
Ion recombination correction $P_{\text{ion}}$ plotted as a function of the renormalized signal. Table 1 shows the best fitting linear parameters for slope and intercept.

A number of parameters could influence $P_{\text{ion}}$. It is well established that $P_{\text{ion}}$ depends on the dose per pulse, and we worked with a modified version of this by referencing our raw signals to the signal for a $10\times 10\;{\text{cm}}^{2}$ field with the chamber set to SAD conditions at depth of dose maximum and 300 V. Other parameters that could influence on $P_{\text{ion}}$ are the energy, the field size, and the chamber (IC10 or A19). Since McEwen^(6)^ studied $P_{\text{ion}}$ for a much larger collection of chambers, our intent was not to confirm these chamber results but to know if separate adjustments to $P_{\text{ion}}$ were needed for each of our chambers. JMP's multivariate analysis shows $P_{\text{ion}}$ depends significantly on the signal and on the energy, but not the field size nor chamber once the effects of signal and energy were taken in to account. Figure 2 shows the $P_{\text{ion}}$ as a function of the renormalized signal with points categorized by energy. The 6 FFF, 10 FFF, and 6/10 MV data were all significantly different from each other. We combined the 6 MV and 10 MV data because these were not significantly different from each other. Figure 2 shows a reasonably linear relation between $P_{\text{ion}}$ and $R^{*}$. We expect the intercept (i.e., $P_{\text{ion}}$) to be 1 when there is no signal. Best‐fit lines, given by Eq. (8), have the slopes, intercepts, 95% HDIs, and coefficients of determination ($R_{2}$) listed in Table 1:

(8) $$P_{ion}=mS^{*}+b$$

Equations (9) and (10) show the relation between corrected and uncorrected signals, S and $R^{*}$, using the fitted parameters. The latter is useful for taking corrected tabulated dose functions and converting to corresponding uncorrected functions.

(9) $$S={m(S^{*})}^{2}+bS^{*}$$

(10) $$S^{*}=\frac{-b+\sqrt{b^{2}+4mS}}{2m}$$

**Table 1:** Slope and intercept parameters for $P_{\text{ion}}$ and the signal $R^{*}$

*Energy*	*m*	*b*	*95% HDI for m*	*95% HDI for b*	*Coefficient of Determination* ($R_{2}$)
10 FFF	1.83E‐4	0.9992	(1.43E‐4, 2.23E‐4)	(0.996, 1.003)	0.931
6 FFF	9.67E‐5	1.0010	(6.54E‐5, 1.28E‐4)	(0.999, 1.003)	0.706
6/10 MV	5.15E‐5	0.9999	(−1.08E−4, 2.10E‐4)	(0.984, 1.016)	0.693

**Figure 3:**
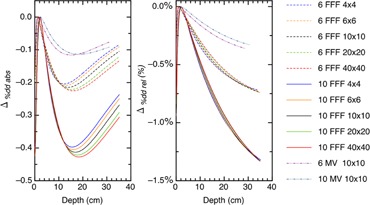
The absolute and relative differences between corrected and uncorrected %dd plotted as a function of energy, depth, and field size.

**Figure 4:**
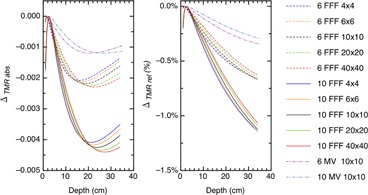
The absolute and relative differences between corrected and uncorrected TMR plotted as a function of energy, depth, and field size.

**Figure 5:**
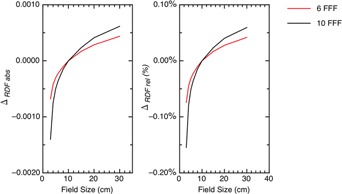
The absolute and relative differences between corrected and uncorrected RDF plotted as a function of energy and field size. The field size is reported by the length of one side of a square field.

**Figure 6:**
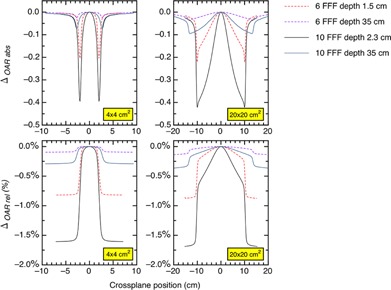
The absolute and relative differences between corrected and uncorrected OAR for $4\times 4\;{\text{cm}}^{2}$ and $20\times 20\;{\text{cm}}^{2}$ fields plotted as a function of energy, depth, and the crossplane position.

The absolute differences and relative differences for the %dd, TMR, and RDF are plotted as a function of the energy, depth, and field sizes in Figs. 3–5. Figure 6 demonstrates the absolute and relative OAR differences for two representative field sizes, $4\times 4\;{\text{cm}}^{2}$ and $20\times 20\;{\text{cm}}^{2}$, taken at depth of dose maximum and at 35 cm depth.

The absolute dose is determined by the quality conversion factor $k_{Q}$ which in turn is determined by the %dd at a depth of 10 cm. Figure 3 shows the $P_{\text{ion}}$ correction to the %dd at a depth of 10 cm is approximately 0.2% and 0.4% for 6 FFF and 10 FFF beams, respectively. Table 2 shows the %dd^*^, %dd, $k_{Q}^{*}$, $k_{Q}$ and $\Delta_{rel\;kQ}$ for the 6 FFF and 10 FFF beams at a depth of 10 cm. Note that we linearly interpolated the $k_{Q}$ values from Table 1 of TG‐519. The relative change in $k_{Q}$ is also the relative change in the absolute dose rate.

**Figure 7:**
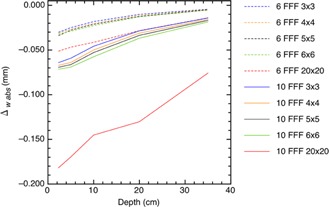
The absolute difference between field widths corrected and uncorrected by $P_{\text{ion}}$ plotted as a function of energy, depth, and field size.

Figure 6 shows there is a maximum change in the OAR located in the penumbra of the fields. The field width is defined by the location of the left and right 50% isodose values and these isodoses lies within the penumbra. In Fig. 7, we plotted the change in field widths, defined in Eq. (7), as a function of the energy, depth, and field size. We focused on small fields because of their importance for 3D conformal planning in SABR techniques, and we included a larger $20\times 20\;{\text{cm}}^{2}$ field for comparison.

**Table 2:** The quality conversion factors for an IC10 chamber with and without the $P_{\text{ion}}$ correction

*Energy*	*%dd* ^*^	*%dd*	$k_{Q}^{*}$	$k_{Q}$	$\Delta_{rel\;kQ}$	$\Delta_{rel\;output}$
6 FFF	63.4	63.2	0.99860	0.99880	0.02%	0.02%
10 FFF	70.8	70.5	0.98936	0.98990	0.05%	0.05%

## IV. DISCUSSION

$P_{\text{ion}}$ corrects an ion chamber's signal to account for ion recombination. A larger signal implies a higher density of ions and a larger probability of ions recombining. This is evident in Fig. 2, demonstrating $P_{\text{ion}}$ increases linearly with signal strength. The three lines describing $P_{\text{ion}}$ for 6 FFF, 10 FFF, and 6/10 MV beams share a common intercept of 1.000 but have slopes that approximately double in magnitude in comparison to one another.

This doubling becomes evident in the difference plots when we compare the curves across energies. For example, the minimum differences for $10\times 10\;{\text{cm}}^{2}$ fields in the %dds are 0.11, 0.21, and 0.42 for the 6/10 MV, 6 FFF, and 10 FFF beams, respectively.

Figures 3 and 4 show that the %dd and TMR behave similarly with the local minimum for the TMR occurring at deeper depths than for the %dd. A minimum occurs because of the interplay of the $P_{\text{ion}}$ and signal strength. These dose functions compare signals to the signal at depth‐of‐dose maximum and, when we correct for ion recombination, we will have a ratio of $P_{\text{ion}}s$. For example, the corrected %dd is

(11) $$\%dd=100\frac{S^{*}P_{ion}}{S_{d_{\text{max}}}^{*}P_{ion,d_{\text{max}}}}$$

At depths near the depth‐of‐dose maximum the $P_{\text{ion}}$ ratio is close to 1.000 and the absolute difference is small. At deep depths, the signal $R^{*}$ is small, while the $P_{\text{ion}}$ ratio is larger. The net result pushes the absolute correction further out in the decimal placing and again the absolute difference is small. There is an intermediate signal value which the $P_{\text{ion}}$ ratio optimally changes the decimal placing in %dd or TMR for maximal effect. The %dd arrives at the optimal signal strength at shallower depths than the TMR because the %dd is modulated by both attenuation and the inverse square law, whereas TMR is only modulated by attenuation.

The OAR behavior is similar to the %dd and TMR behavior, except the signal is changed by moving laterally in the crossplane and the optimal signal for a maximum absolute difference occurs in the penumbra. One extra feature for the profile curves exhibit is the depth dependence to the differences. As the depth increases, the magnitude of the absolute difference decreases. This occurs because the $P_{\text{ion}}$ ratio compares two $P_{\text{ion}}s$ at the same depth. The corrected OAR is

(12) $$OAR(x,FS)=\frac{S^{*}P_{ion}}{S_{CAX}^{*}P_{ion,CAX}}$$

where the *CAX* subscript denotes values taken on the central axis at the same depth. The $P_{\text{ion}}$ ratio is a maximum at the depth‐of‐dose maximum because the signal on the central axis is close to 100 and drops to the optimal value in the penumbra. At another depth, such as 35 cm, the $P_{\text{ion}}$ ratio is much closer to 1.000 because the central axis signal and penumbra signals are both small and have nearly the same $P_{\text{ion}}s$.

The relative changes to the dosimetric functions are more easily understood because these involve relative change to the $P_{\text{ion}}$ ratio. This change is largest at the deepest depths and/or in the penumbras of the fields.

The TMR has one additional subtly worth addressing. When we measure the TMR, we typically measure with the chamber at the isocenter and add water to increase the depth. At depth‐of‐dose maximum, the signal $R^{*}$ will be close to 100 and the addition of water attenuates this signal accordingly. In reality, this measurement is infrequently done and we derive the TMR curves from the %dd curves. This formalism calculates the TMR at depth, d, by calculating it at a point that is at (100 + d) cm from the source. This changes the magnitude of the signals to be used for the $P_{\text{ion}}$ correction, and we need to apply the correct inverse square law and account for other scattering effects. For example, if we measured the TMR for a 10 FFF beam, with field size $10\times 10\;{\text{cm}}^{2}$ and at a depth of 20 cm, our measured signals would be 57.2 at depth and 100 at depth‐of‐dose maximum. If we calculated the same TMR, the calculated signal would be 38.6 at depth and 67.8 at depth‐of‐dose maximum. The $P_{\text{ion}}$ correction is different between measured and calculated because of the difference in the signal magnitudes.

The RDF compares the signal for a given field size to the signal for a $10\times 10\;{\text{cm}}^{2}$ field. The function is corrected by a ratio of $P_{\text{ion}}s$, but the degree of variation in this $P_{\text{ion}}$ ratio is much smaller because it compares two signals that are within ±10 units of each other on our reference scale. The slope for $P_{\text{ion}}$ is of the order $10^{-4}$ per unit and hence we would expect absolute changes on the order of $10^{-3}$. We see this in Fig. 5.

The change in the absolute output is 0.02% for 6 FFF beams and 0.05% for 10 FFF beams. Although negligible in clinical applications, it was important to confirm the change.

The $P_{\text{ion}}$ correction reduces the OAR. This technically narrows the radiation field. The OAR reduction is greatest at the depth‐of‐dose maximum but, for field sizes $6\times 6\;{\text{cm}}^{2}$ or smaller, the change in field width is less than 0.03 and 0.07 mm for 6 FFF and 10 FFF beams, respectively. This is not significant because TG‐142^(14)^ uses a 2 mm tolerance for field size QA.

## V. CONCLUSIONS

We presented a method of accounting for charge recombination effects in flattening filter‐free and flattened beams that exploits the linear variation of $P_{\text{ion}}$ with signal strength. The procedure is flexible in that it can be applied to both single‐voltage measurements and dosimetric functions derived from other dosimetric functions. $P_{\text{ion}}$ corrections on %dd, TMR, and OAR are up to 0.4% for 6/10 MV, 0.8% for 6 FFF, and 1.6% for 10 FFF beams. Corrections on RDF, field size, and absolute dose rate are detectable, but insignificant, in clinical settings.

## References

[1] Xiao Y , Fry SF , Popple R , et al. Flattening filter‐free accelerators: a report from the AAPM Therapy Emerging Technology Assessment Work Group. J Appl Clin Med Phys. 2015;16(3):12–29. 10.1120/jacmp.v16i3.5219 PMC5690108 26103482

[2] Chang Z , Wu Q , Adamson J , et al. Commissioning and dosimetric characteristics of TrueBeam system: composite data of three TrueBeam machines. Med Phys. 2012;39(11):6981–7018. 23127092 10.1118/1.4762682

[3] Lang S , Hrbacek J , Leong A , Klöck S . Ion‐recombination correction for different ionization chambers in high dose rate flattening filter‐free photon beams. Phys Med Biol. 2012;57(9):2819–27. 22510780 10.1088/0031-9155/57/9/2819

[4] Wang Y , Easterling SB , Ting JY . Ion recombination corrections of ionization chambers in flattening filter‐free photon radiation. J Appl Clin Med Phys. 2012;13(5):262–68. 10.1120/jacmp.v13i5.3758 PMC5718222 22955642

[5] Kry SF , Popple R , Molineu A , Followill DS . Ion recombination correction factors (P(ion)) for Varian TrueBeam high‐dose‐rate therapy beams. J Appl Clin Med Phys. 2012;13(6):318–25. 10.1120/jacmp.v13i6.3803 PMC5718527 23149774

[6] McEwen MR . Measurement of ionization chamber absorbed dose k_Q_ factors in megavoltage photon beams. Med Phys. 2010;37(5):2179–93. 20527552 10.1118/1.3375895

[7] Kim S , Huh H , Choi S , Min CH , Shin D , Choi J . Polarity and ion recombination correction factors of a thimble type ionization chamber with depth in water in the megavoltage beams. J Radiat Prot. 2009;34(2):435–43.

[8] El‐Hafez AI , Shousha HA , Zaghloul MS , Abou Zeid MA . Influence of field size, depth, nominal dose rate and stem length on ion recombination correction factor in therapeutic photon beam. J Am Sci. 2011;7(3):206–13.

[9] Almond PR , Biggs PJ , Coursey BM , et al. AAPM's TG‐51 protocol for clinical reference dosimetry of high‐energy photon and electron beams. Med Phys. 1999;26(9):1847–70. 10505874 10.1118/1.598691

[10] McEwen M , DeWerd L , Ibbott G , et al. Addendum to the AAPM's TG‐51 protocol for clinical reference dosimetry of high‐energy photon beams. Med Phys. 2014;41(4):041501. 24694120 10.1118/1.4866223 PMC5148035

[11] Nisbet A and Thawaites DI . Polarity and ion recombination correction factors for ionization chambers employed in electron beam dosimetry. Phys Med Biol. 1998;43(2):435–43. 9509537 10.1088/0031-9155/43/2/016

[12] Boag JW and Currant J . Current collection and ionic recombination in small cylindrical ionization chambers exposed to pulsed radiation. Br J Radiol. 1980;53(629):471–78. 7388281 10.1259/0007-1285-53-629-471

[13] Weinhous MS and Meli JA . Determining Pion, the correction factor for recombination losses in an ionization chamber. Med Phys. 1984;11(6):846–49. 6513890 10.1118/1.595574

[14] Klein EE , Hanley J , Bayouth J , et al. Task Group 142 report: quality assurance of medical accelerators. Med Phys. 2009;36(9):4197–212. 19810494 10.1118/1.3190392

